# Acceptance and Commitment Therapy for Children with Special Health Care Needs and Their Parents: A Systematic Review and Meta-Analysis

**DOI:** 10.3390/ijerph18158205

**Published:** 2021-08-03

**Authors:** Arpita Parmar, Kayla Esser, Lesley Barreira, Douglas Miller, Leora Morinis, Yuen-Yu Chong, Wanda Smith, Nathalie Major, Paige Church, Eyal Cohen, Julia Orkin

**Affiliations:** 1 Department of Paediatrics, The Hospital for Sick Children, Toronto, ON M5G 1X8, Canada; arpita.parmar@sickkids.ca (A.P.); kayla.esser@sickkids.ca (K.E.); lesleybarreira@gmail.com (L.B.); eyal.cohen@sickkids.ca (E.C.); 2 Child Health Evaluative Sciences, SickKids Research Institute, The Hospital for Sick Children, Toronto, ON M5G 1X8, Canada; 3 Department of Psychiatry and Behavioural Neurosciences, McMaster University, Hamilton, ON LS8 4L8, Canada; doug.miller@sickkids.ca (D.M.); smithwa@mcmaster.ca (W.S.); 4 Institute of Health, Policy, Management & Evaluation, Univeristy of California San Francisco, San Francisco, CA 94143, USA; leora.morinis@gmail.com; 5 The Nethersole School of Nursing, The Chinese University of Hong Kong, Hong Kong 999077, China; conniechong@cuhk.edu.hk; 6 Department of Pediatrics, Children’s Hospital of Eastern Ontario, Ottawa, ON K1H 8L1, Canada; nmajor@cheo.on.ca; 7 Divison of Neonatology, Sunnybrook Health Sciences Centre, Toronto, ON M4N 3M5, Canada; paige.church@sunnybrook.ca; 8 Institute of Health, Policy, Management and Evaluation, Dalla Lana School of Public Health, University of Toronto, Toronto, ON M5T 3M6, Canada; 9 Department of Paediatrics, Faculty of Medicine, University of Toronto, Toronto, ON M5T 1P7, Canada

**Keywords:** acceptance and commitment therapy, children with special health care needs

## Abstract

**Context:** Acceptance and Commitment Therapy (ACT) is an emerging treatment for improving psychological well-being. **Objective:** To summarize research evaluating the effects of ACT on psychological well-being in children with special health care needs (SHCN) and their parents. **Data Sources:** An electronic literature search was conducted in PubMed, Web of Science, Ovid/EMBASE and PsycINFO (January 2000–April 2021). **Study Selection:** Included were studies that assessed ACT in children with SHCN (ages 0–17y) and/or parents of children with SHCN and had a comparator group. **Data Extraction:** Descriptive data were synthesized and presented in a tabular format, and data on relevant outcomes (e.g., depressive symptoms, stress, avoidance and fusion) were used in the meta-analyses to explore the effectiveness of ACT (administered independently with no other psychological therapy) compared to no treatment. **Results:** Ten studies were identified (child (7) and parent (3)). In children with SHCN, ACT was more effective than no treatment at helping depressive symptoms (standardized mean difference [SMD] = −4.27, 95% CI: −5.20, −3.34; *p* < 0.001) and avoidance and fusion (SMD = −1.64, 95% CI: −3.24, −0.03; *p* = 0.05), but not stress. In parents of children with SHCN, ACT may help psychological inflexibility (SMD = −0.77, 95% CI: −1.07, −0.47; *p* < 0.01). **Limitations:** There was considerable statistical heterogeneity in three of the six meta-analyses. **Conclusions:** There is some evidence that ACT may help with depressive symptoms in children with SHCN and psychological inflexibility in their parents. Research on the efficacy of ACT for a variety of children with SHCN and their parents is especially limited, and future research is needed.

## 1. Introduction

### 1.1. Children with Special Health Care Needs (SHCN)

In recent decades, there has been a dramatic shift in the landscape of pediatrics: with advanced screening, detection and treatment modalities, what were once considered to be largely fatal or life-threatening diseases can now be managed for the long-term [1]. As a result of this epidemiologic shift, millions of children worldwide are now facing a new reality: living with special health care needs (SHCN) [1]. Children with SHCN are “those who have or are at increased risk for a chronic physical, developmental, behavioral, or emotional condition and who also require health-related services of a type or amount beyond that required by children generally” [2]. Children with SHCN may have only one condition, or both a chronic physical and emotional condition [1,3] such as co-existing depression with type I diabetes [4]. 

### 1.2. Psychological Difficulties Experienced by Children with SHCN and Their Parents

It is estimated that 17–23% of children with SHCN [4,5,6] are affected by depression, in contrast to 5–9% of healthy children [7,8,9]. The increased prevalence of depression in children with SHCN may be attributed to difficulties adapting to their daily life with a medical condition, which may include frequent clinic and hospital visits, use of medications and/or medical devices, missed school days and social functions [3]. Additionally, parents of children with SHCN have been shown to be negatively impacted by caring for children with SHCN [10], with increased rates of stress, [11] anxiety, and depression compared to parents of otherwise healthy children [12,13]. Parents of children with SHCN have described feeling fearful, alone and powerless about their child’s diagnosis and uncertain about their child’s future health or overall future [11]. Various aspects of the child’s illness may negatively impact parent well-being including demanding treatment regimens, an increase in responsibilities (e.g., attending medical appointments) and a shift in lifestyle (e.g., less time at work and more at home with the child) [10,11].

### 1.3. Psychological Interventions and Their Current Stage of Knowledge

Interventions to support the psychological well-being of children with SHCN and their families are limited [14,15,16]. Only two drugs for depression and anxiety (fluoxetine and citalopram) are currently approved for use by the United States Food and Drug Administration and the Canadian Drug Safety Agency [17,18], both of which have limited efficacy [19]. Therefore, behavioral therapies, such as Cognitive Behavioural Therapy (CBT), are often the first choice of treatment for depression and anxiety [20]. CBT is generally short-term and focused on helping individuals cope with negative thinking or a specific problem, that is labelled as a cognitive distortion [21]. After successful treatment with CBT, individuals learn how to identify and change thinking patterns that negatively influence their feelings and behavior [21]. However, the evidence for CBT in pediatric depression (ages <18 years) is limited as a meta-analysis that included six studies shows that over 80% of the included studies (5/6) reported small model-based mean effect-sizes ranging between 0.13–0.27 [22]. 

Research on other treatments for pediatric depression are strongly warranted as negative outcomes associated with untreated depression can include poor school performance, poor quality of life, substance abuse and suicide [23,24,25,26,27]. Additionally, approximately 5 million children and adolescents worldwide receive treatment for depression every year [28], which has a substantial economic burden as it is the most expensive pediatric condition to treat with an estimated annual cost of over 10 billion dollars [28]. 

### 1.4. Acceptance and Commitment Therapy

A newer empirically supported behavioural therapy that focuses on improving psychological well-being is Acceptance and Commitment Therapy (ACT), which incorporates mindful attention and the practices of acceptance and values-based action to cope with difficult experiences [29]. ACT differs from CBT, which helps individuals cope with and correct negative thoughts (such as cognitive distortions) [21]. Instead, ACT focuses on changing one’s relationship to their thoughts and emotions, with symptom reduction occurring as a by-product [29]. 

**Figure 1:**
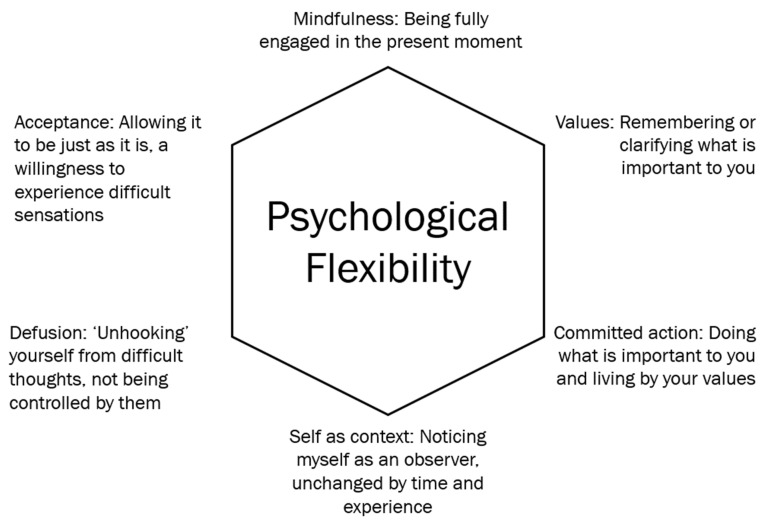
The framework of Acceptance and Commitment Therapy.

ACT incorporates practices of mindful acceptance to improve psychological flexibility [29]. Psychological flexibility is the ability to contact the present moment and inner thoughts and feelings without defense, while persisting in the pursuit of goals and values through committed action [29]. Psychological flexibility is established through six interrelated processes: cognitive defusion, acceptance, committed action, values, contact with the present moment, and self-as-context [29] (See Figure 1). Cognitive defusion is the ability to create distance from thoughts and feelings (defusing from them) in order to prevent them from controlling behavior [30]. This is linked to acceptance, the process of making space for thoughts and feelings as they arise, without trying to alter or resist them [30]. Acceptance of thoughts and feelings allows for the engagement in committed action, which is moving towards what is important and meaningful despite the presence of potentially distressing thoughts and feelings [30,31]. Committed actions are driven by values, or “chosen life directions” [31]. Values could be related to what a person stands for, or what qualities they want to embody in life [31]. The last two ACT principles are contact with the present moment and self-as-context. The first refers to being engaged and aware in the present moment and the surrounding environment [30,31]. The second, self-as-context, is also referred to as “the observing self”, which focuses on taking a step back and noticing thoughts and feelings [31]. This ability to take a flexible perspective is central to ACT and facilitates the other 5 processes [30].

ACT encourages people to embrace their thoughts and feelings rather than avoiding them. According to ACT, the two core processes at the root of distress are cognitive fusion and experiential avoidance [31]. Cognitive fusion is the process of getting caught up in thoughts and feelings, which may dominate behaviour (e.g., fusing with the idea that children with physical disabilities cannot play sports) [31]. Experiential avoidance is the process of suppressing negative thoughts, which may provide short-term relief, but can lead to long-term psychological suffering [31]. Avoidance and fusion can increase psychological distress and prevent living in the present moment [31]. ACT has also been shown to foster resilience [32,33], which is the ability to adapt to adversity [34]. ACT may help improve resilience by improving psychological flexibility, so individuals have a less rigid and more adaptive response to challenging emotions and experiences [35]. With ACT, adolescents with depression have shown improved resilience by learning to accept difficult emotions rather than avoiding them, and not fusing with a ‘depressed identity’ by learning that they are not defined by their challenging emotions [33]. 

### 1.5. Acceptance and Commitment Therapy for Children with SHCN and Their Parents

ACT may also be well-suited for children with SHCN and their parents, given that their overwhelming feelings and challenges are likely a reflection of an unfortunate reality rather than a cognitive distortion [36]. There is emerging literature on the effectiveness of ACT for children. One systematic review reported that the use of ACT in children was increasing and it was effective in improving psychological flexibility [37]. However, authors reported methodological inadequacies identified in the research reviewed and also included non-peer reviewed articles [37]. A second systematic review reported ACT significantly improved symptoms of anxiety and depression in children when compared to usual treatment/waitlist controls [38]. However, this review also included studies with healthy children, making it difficult to generalize findings to children with SHCN. Both reviews are further limited because they: (1) did not focus solely on children (<18 years of age) as they included studies with young adults (ages 18–21 years of age) and (2) assessed the efficacy of ACT in conjugation with another therapy (e.g., CBT). More research on the efficacy of ACT as an independent therapy in parents of children with SHCN is warranted [39,40], as a commitment to family-centered care in pediatrics and because evidence on the efficacy of interventions is lacking [41]. 

Thus, the objective of this systematic review and meta-analysis is to provide a comprehensive summary of the current evidence on the efficacy of ACT on psychological outcomes and ACT-related process variables (e.g., avoidance and fusion) in children with SHCN and parents of children with SHCN. We also aim to evaluate the quality of included research studies, comment on the generalizability of their findings, and highlight steps for future research.

## 2. Methods

### 2.1. Search Strategy

An electronic literature search was completed in PubMed, Web of Science, Ovid/EMBASE and PsycINFO from January 2000 through April 2021. This period was selected because the official ACT treatment manual was published in 1999 [42], which led to an increase in its use both clinically and in research [43]. Search terms were: “Acceptance and Commitment Therapy” AND “children with special health care needs”; “children”; “adolescents”; “pediatrics”; “parents”; “caregivers”. Articles were selected in a two-step process independently by two reviewers (AP and KE). Each reviewer assessed the titles and relevant abstracts, then conducted a full-text review of the relevant articles based on the inclusion criteria. Consensus for studies included for review was achieved by discussion between the authors and adjudication by a third reviewer (JO).

### 2.2. Eligibility Criteria

Inclusion criteria are studies that:

1. Assessed the efficacy of ACT on psychological outcomes (e.g., depressive symptoms, interpersonal problems, psychological flexibility) and/or ACT-related process variables (e.g., avoidance and fusion) on children with SHCN (0 to <18 years) and/or the parents of children with SHCN.
2. Administered ACT independently and not in combination with another therapy or treatment (e.g., CBT).
3. Included a comparison group (e.g., treatment as usual, waitlist control).
4. Were published from January 2000–April 2021.
5. Were written in English.
6. Were original peer-reviewed full-length articles.

### 2.3. Data Extraction

Study characteristics and data were extracted and entered into tables (see Table 1). The structure of this systematic review followed the Preferred Reporting Items for Systematic Reviews and Meta-Analyses (PRISMA) guidelines [44].

### 2.4. Risk of Bias

The Cochrane Collaboration’s tool for assessing risk of bias in randomized trials was used independently by two reviewers to evaluate the risk of bias of the included studies. Each component was rated as “high risk”, “low risk” or “unclear risk” and a summary assessment of risk of bias was provided within the trial based on reporting guidelines [45,46]. 

### 2.5. Statistical Analysis and Data Synthesis

Review Manager (RevMan) version 5.3 (London, UK) [47] was used to conduct the meta-analyses. Extracted data were descriptively presented in tabular form and the results outcomes (depression, stress, avoidance and fusion, anxiety, and psychological flexibility) were visualized through forest plots. Effect sizes were calculated for rating scales with continuous data by assessing the standard mean difference (SMD) with 95% confidence intervals (CIs). For each outcome, the SMD across the identified studies was summarized through a meta-analysis, using a random-effects model for continuous data by measuring the change in scores (pre- and post-ACT or pre- and post-no treatment for controls). We only compared outcomes directly after the ACT intervention as follow-up lengths varied across the studies. When the standard deviation (SD) of the difference was not provided, corresponding authors were contacted for these data points. If no response was provided after two contact attempts, the following formula was used for the calculation of the SD of the difference from the SD given at each time point: SD of the difference = √(SD1^2^ + SD2^2^ − 2r × SD^1^ × SD^2^) [48] and r was estimated to be 0.7 [49].

A negative SMD indicates lower scores (better outcomes) in the exposed group (ACT) and a positive SMD indicates lower scores in the unexposed group (control). The percentage of variability attributed to heterogeneity between the studies was assessed using the I2 statistic. Using the Cochrane Handbook guidelines for systematic reviews, an I2 of 0% to 40% represented possibly unimportant heterogeneity, 30% to 60% moderate heterogeneity, 50% to 90% substantial heterogeneity, and 75% to 100% considerable heterogeneity [45]. Statistical significance was set at a *p* value of ≤ 0.05 and significant associations and effect sizes (small d = 0.2–0.4, medium d = 0.5–0.7, large d ≥ 0.8) [50] were reported if available. 

## 3. Results

### 3.1. Search Results

**Figure 2:**
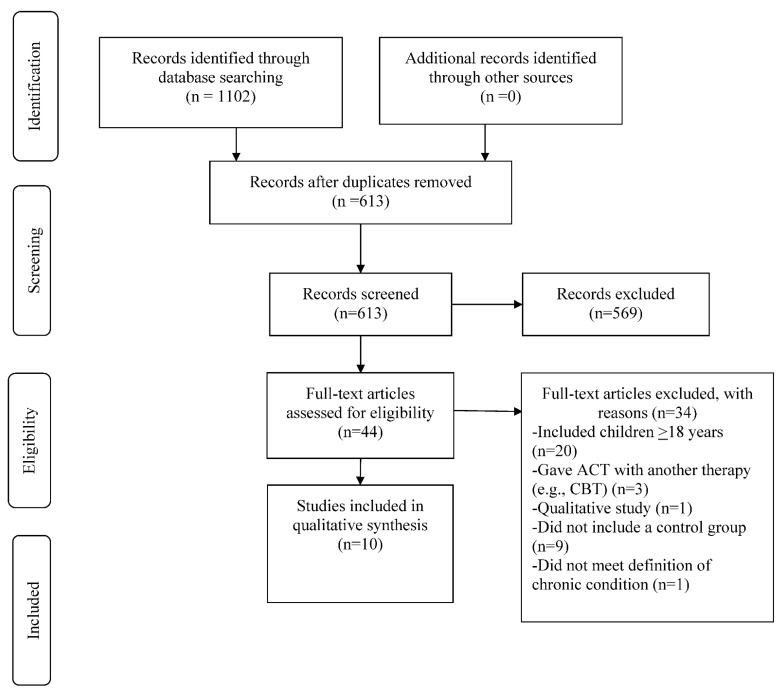
PRISMA Flow Diagram of Study Selection.

The initial search identified 1102 titles. After duplicates were removed, 613 titles and abstracts were reviewed and 569 were excluded because of irrelevance based on inclusion criteria. 44 articles were downloaded for further consideration and 10 studies met all inclusion criteria. The PRISMA flowchart is presented in Figure 2.

### 3.2. Study Characteristics

Table 1 summarizes study characteristics of the 10 relevant studies. Four studies were randomized controlled trials [37,51,52,53], three were quasi-experimental pre-post designs with random assignment [54,55,56], and three were pre-post with control groups and random assignment [57,58,59]. Five studies were conducted in Iran [54,55,56,57,58], two in Australia [51,60], one in Sweden and Australia (one article with a study cohort in each country) [52], and one in Hong Kong [53] and one in the USA [59]. ACT was administered via weekly sessions (average of 8 sessions) with a trained psychologist, or a trained nurse supervised by a psychologist. The efficacy of ACT was assessed in the following chronic conditions: children with Type I and II diabetes (2 studies) [57,58], anxiety (3 studies) [51,54], chronic pain [55], depressive symptoms [52] and psychological problems (measured by the Strengths and Difficulties Questionnaire) [52]. ACT was also assessed in studies of parents of children with asthma [53], autism [59], and hearing impairments or deafness [56]. The studies included in the meta-analysis on children with SHCN assessed the effect of ACT on the following outcomes: avoidance and fusion (3 studies) [51,52], depression (2 studies) [52,57] and stress (2 studies) [52,58]. The meta-analysis on parents of children with SHCN assessed the effect of ACT on the following outcomes: psychological flexibility (2 studies), depression (2 studies) and anxiety (2 studies). A descriptive qualitative synthesis was presented for the two studies in children with SHCN that could not be included in the meta-analysis, which did not assess depression, stress or avoidance and fusion as an outcome. 

### 3.3. Risk of Bias Assessment

**Figure 3:**
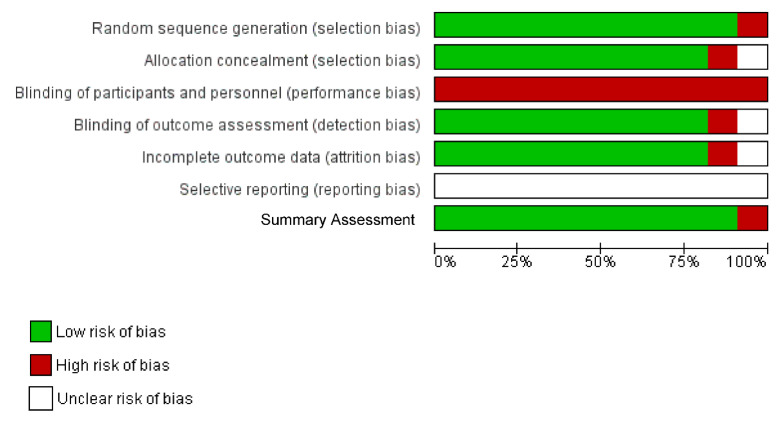
Risk of bias graph: review authors’ judgements about each risk of bias item presented as percentages across all included studies.

**Figure 4:**
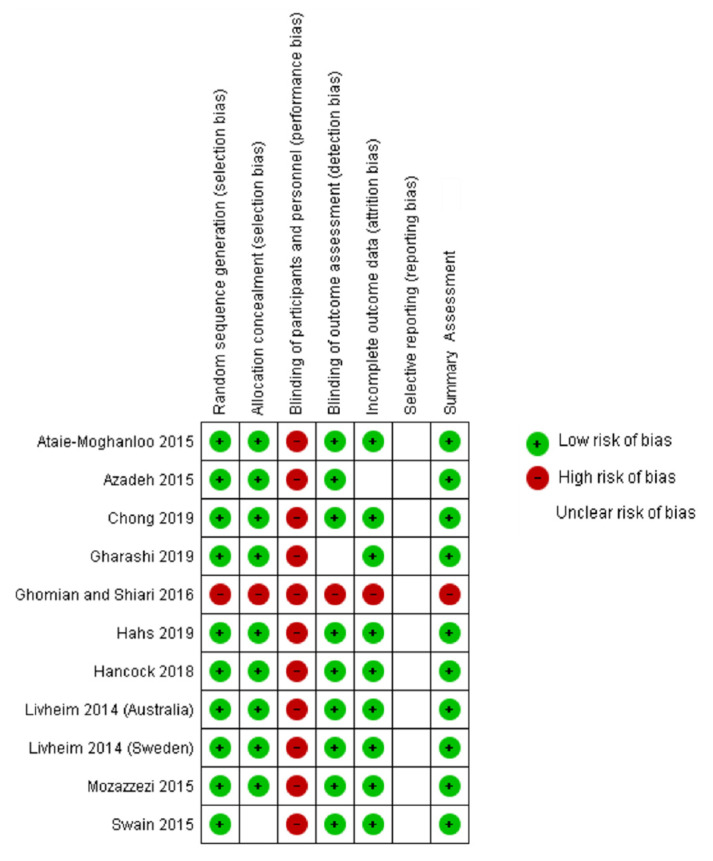
Risk of bias summary: review authors’ judgements about each risk of bias item presented as percentages across all included studies.

Of the 10 included studies (1 manuscript included two trials), 8 were classified as having a low risk of bias [51,52,53,54,57,58] and 1 was classified as having a high risk of bias [55], on the summary risk of bias assessments. Details of the risk of bias are in Figure 3 and summary scores are in Figure 4.

### 3.4. Meta-Analysis of Reported Outcomes

#### 3.4.1. Children with SHCN

Three individual meta-analyses were performed on studies reporting outcomes of depressive symptoms, stress and avoidance and fusion in children with SHCN.

- *Depressive Symptoms*

**Figure 5:**
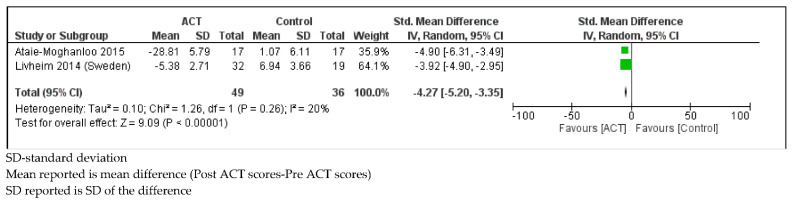
Forest plot comparing depression scores between children with SHCN who received ACT and children with SHCN who received no treatment.

Children with SHCN who received ACT had lower depression scores (measured by the Reynolds’ Child Depression Scale) compared to controls (SMD = −4.27, 95% CI: −5.20, −3.34; *p* < 0.001) with low heterogeneity (I^2^ = 20%; *p* = 0.26; Figure 5) [52,57].

- *Stress*

**Figure 6:**
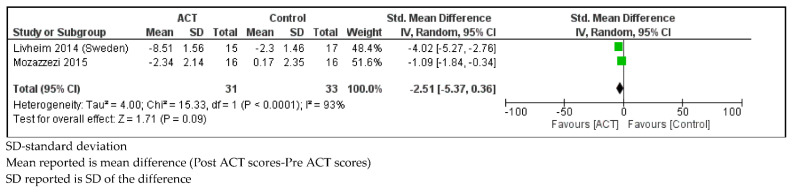
Forest plot comparing stress scores between children with SHCN who received ACT and children with SHCN who received no treatment.

Children with SHCN who received ACT had lower point estimates of stress scores (measured by the Perceived Stress Scale) compared to controls, however the confidence intervals were wide, and differences did not meet threshold for significance (SMD = −2.51, 95% CI: −5.37, 0.36; *p* = 0.09) with considerable statistical heterogeneity (I^2^ = 93%; *p =<* 0.001; Figure 6) [52,58].

- *Avoidance and Fusion*

**Figure 7:**
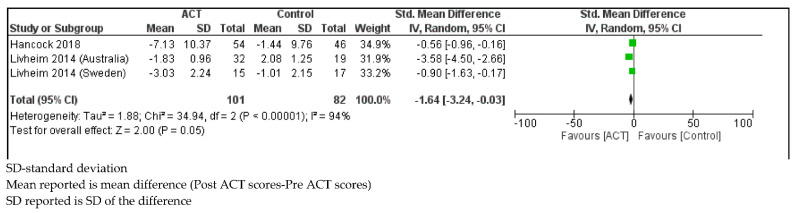
Forest plot comparing avoidance and fusion scores between children with SHCN who received ACT and children with SHCN who received no treatment.

Children with SHCN who received ACT showed a decrease in avoidance and fusion scores compared to controls (SMD = −1.64, 95% CI: −3.24, −0.03; *p* = 0.05) with considerable statistical heterogeneity (I^2^ = 94%; *p* < 0.001; Figure 7) [51,52].

#### 3.4.2. Parents of Children with SHCN

Three individual meta-analyses were performed on studies reporting outcomes of depressive symptoms, anxiety and psychological flexibility in parents of children with SHCN.

- *Depressive Symptoms*

**Figure 8:**
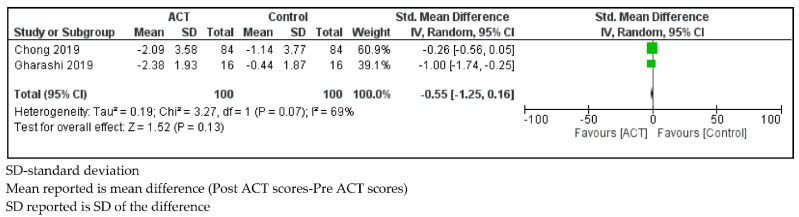
Forest plot comparing Depression, Anxiety and Stress Scale-21 (Depression) scores between parents of children with SHCN who received ACT and parents of children with SHCN who received no treatment.

Parents of children with SHCN who received ACT showed no change in Depression, Anxiety and Stress Scale-21 (DASS-21) depression scores compared to controls (SMD = −0.55, 95% CI: −1.25, −0.47; *p* < 0.01) with substantial statistical heterogeneity (I^2^ = 69%; *p* = 0.07; Figure 8).

- *Anxiety*

**Figure 9:**
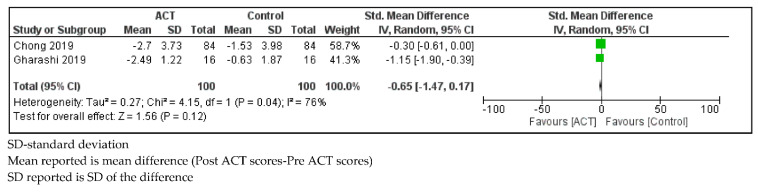
Forest plot comparing Depression, Anxiety and Stress Scale-21 (Anxiety) scores between parents of children with SHCN who received ACT and parents of children with SHCN who received no treatment.

Parents of children with SHCN who received ACT showed no change in DASS-21 anxiety scores compared to controls (SMD = −0.65, 95% CI: −1.47, 0.17; *p* = 0.12) with considerable statistical heterogeneity (I^2^ = 76%; *p* = 0.04; Figure 9).

- *Psychological Flexibility*

**Figure 10:**
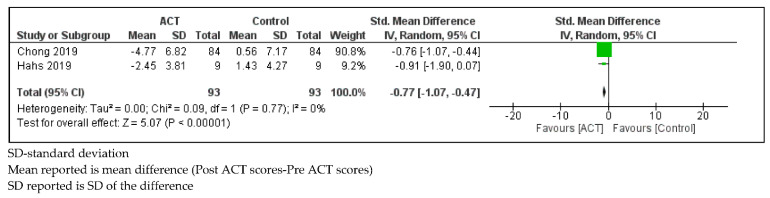
Forest plot comparing acceptance and action questionnaire-II scores between parents of children with SHCN who received ACT and parents of children with SHCN who received no treatment.

Parents of children with SHCN who received ACT showed a decrease in acceptance and action questionnaire-II (AAQ-II) scores compared to controls (SMD = −0.77, 95% CI: −1.07, −0.47; *p* < 0.01) with low statistical heterogeneity (I^2^ = 0%; *p* = 0.77; Figure 10).

### 3.5. Qualitative Synthesis of Systematic Review

#### Children with SHCN

- *Behaviour and Interpersonal Problems*

Ghomian and Shairi reported improvements in externalizing and aggressive behaviour difficulties measured by the Child Behavior Checklist (CBCL) in children with chronic pain receiving ACT compared to those not receiving ACT, which was sustained in 5-month follow-up visits [61]. In contrast, Swain et al. reported no change in CBCL total problems scores in children with anxiety disorders post-ACT [60]. Further, Azadeh et al. reported a decrease in interpersonal problems (e.g., problems with sociability) with a medium effect size (Cohen’s d = 0.71) in teenagers with social anxiety disorder [54].

- *Anxiety*

Hancock et al. reported improvements for children with a diagnosis of anxiety disorder on the Anxiety Disorders Interview Schedule with large effect sizes (Cohen’s d = 2.59), and on the Multidimensional Anxiety Scale for Children with medium effect sizes (Cohen’s d = 0.53), which were maintained at the 3-month follow-up [51]. Hancock et al. also found a significant reduction in clinical severity rating of anxiety, measured through clinical interviews [51]. At post-ACT intervention, 37% of participants no longer met criteria for an anxiety disorder and the effects were sustained at the 3-month follow-up [51]. Additionally, Livheim et al. reported improvements in anxiety measured by the DASS-21 with large effect size (Cohen’s d = 0.80) in teenagers with depressive symptoms [52].

- *Psychological flexibility*

Azadeh et al. reported an improvement in psychological flexibility (measured by AAQ-II) (Cohen’s d = 0.61) [54].

## 4. Discussion

### 4.1. Key Findings

This meta-analysis and systematic review identified 10 peer-reviewed articles assessing the effects of ACT in children with SHCN (7 articles) and their parents (3 articles). Our meta-analyses suggest that ACT may help depressive symptoms and avoidance and fusion behavior in children with SHCN, as well as improve psychological flexibility in parents of children with SHCN. Findings from the qualitative synthesis of the systematic review suggest that ACT may also be effective for improving anxiety, psychological flexibility, behavior and interpersonal problems in children with SHCN. 

The findings from our meta-analyses suggest that ACT may help depressive symptoms in children with SHCN. This differs from findings from a meta-analysis on three studies with a similar age group of children and youth with SHCN with depression (e.g., co-existing depression with diabetes) that showed ACT did not help depressive symptoms (SMD = 1.02, 95% CI = −0.11, 2.15, *p* = 0.08, I^2^ = 86%) [62]. This difference may be caused by the use of different standard deviation values and age cut-offs. Further exploration is needed to understand how ACT may translate into clinical care for children with SHCN with co-existing depression. 

In addition, our meta-analyses revealed that ACT was effective in reducing avoidance and fusion scores in children with SHCN. This was an expected finding, as ACT approaches focus on improving avoidance and fusion, which can help with behavioral and psychological flexibility [29,63]. It has also been suggested that reductions in avoidance and fusion mediate the improvements in outcomes such as symptoms of depression and anxiety, seen with an ACT intervention [64]. 

Further, the qualitative synthesis of the systematic review showed that in two studies, children with SHCN had improvements in anxiety symptoms after ACT [51,52]. However, the reasons for the demonstrated decrease in anxiety symptoms following ACT are unclear. It may be speculated that since ACT teaches children that anxious thoughts are just thoughts, patients may be able to face them with mindful acceptance rather than resistance and may feel more at ease [65]. 

One study reported ACT helped challenging behavior in children with SHCN [61], however another study did not report any improvements [60] for reasons that are unclear. It is possible that ACT may help challenging behavior by calming children down through mindfulness activities and encouraging committed actions in line with the child’s values [66]. One study also reported improved psychological flexibility in teenagers with social anxiety disorder [54]. Psychological flexibility is the central component of ACT (see Figure 1) which allows individuals to adapt to challenging situations in life, while being aware, open and committed to behaviors that are in line with their values [67]. This flexible approach is important in coping with conditions such as depression and anxiety, as psychological flexibility may be absent in many forms of psychopathology [67].

Finally, our findings from the meta-analysis on parents of children with SHCN, which included two studies on parents of children with asthma and autism, highlighted that ACT was effective at helping psychological flexibility [53,59]. These findings are similar to a recent study that assessed the efficacy of ACT on psychological flexibility in parents of youth with chronic pain but was not included in this review/meta-analysis because it did not include a comparator group [68]. However, our findings on parents of children with SHCN did not show improvements in depressive symptoms and anxiety in parents. For reasons that are unclear, these findings differ from a recent meta-analysis that shows ACT is effective at improving depression (SMD = 0.52, 95% CI = 0.33, 0.71, *p* < 0.001, I^2^ = 40%) in adults with medical conditions (e.g., cancer) [62]. 

### 4.2. Recommendations for Future Research

This review summarized the potential efficacy of ACT on the psychological outcomes of parents and their children with SHCN. Despite limited evidence, our review shows that ACT may be an effective therapy to add to the armamentarium for depressive symptoms for children with SHCN. Further research with larger samples, ensuring methodological rigor and a more diverse population of children with SHCN are needed in order to understand the effects of ACT for children with SHCN. More importantly, the role of ACT in fostering parental psychological flexibility is well noted in our review. There has been a growing body of knowledge discussing the roles of parental psychological flexibility in fostering parental psychological well-being and their child’s health [69,70]. Interventions to improve parental psychological well-being are limited [41] but are needed as they may help support the parent’s caregiving abilities and consequently their child’s health [71]. The relationship between psychological inflexibility, poor parent psychological well-being and their child’s health may be explained by the Family Stress Model [35,72], which highlights that external stressors (e.g., a child’s illness) may directly affect the parent’s well-being (e.g., by having a depressed mood) which affects their ability to care for their child with SHCN (e.g., administer complex treatment regimens) [35,73]. One of the studies included in our review demonstrated that ACT could be delivered by trained health care professionals (e.g., frontline nurses) to parents of children with asthma, integrated with evidence-based asthma management program, and ultimately demonstrated mutual health benefits for parent-child dyads [53]. This evidence may pave the pathways for future studies in exploring the efficacy of ACT-based disease management programs for families of children with SHCN or integrating ACT components when providing patient care (e.g., communication or brief counselling) to these vulnerable and distressed families. 

Future research exploring the efficacy for ACT in parents of children with SHCN is warranted as a previous meta-analysis highlighted that these parents have a greater risk of depression (SMD = 0.31, 95% CI: 0.24–0.39; *p* < 0.001, I^2^ = 69%) and anxiety (SMD = 0.42, 95% CI: 0.24–0.60; *p* < 0.001, I^2^ = 78%) when compared with parents of healthy children [13]. Psychological conditions are the leading cost of health care spending in adults [74] and parental distress of caring for a child with a medical condition has a significant impact on the adult health care system [75]. Improved parent mental health is an important priority for pediatric health teams who are dedicated to promoting family-centered care [76].

### 4.3. Limitations of This Systematic Review and Meta-Analysis

Limitations of the reviewed studies include short follow-up periods (e.g., 3–6 months) which inhibited the ability to assess for the sustained effects of ACT. Additionally, the included studies were mostly of children with psychosocial conditions, making it difficult to generalize findings to a wider range of children with SHCN with a variety of chronic medical conditions. In addition, only one study from the United States of America met the inclusion criteria, so findings may not be generalizable to populations in North America, while the rest of the included studies were completed in Iran, Australia, Sweden and Hong Kong. Another limitation of the included studies was that socioeconomic and demographic characteristics of the child and family (e.g., income, parental education level) were not accounted for, and future research should assess how these variables affect ACT as a treatment. The studies were also mostly on children within the adolescent age group (10–18 years) so findings may not be generalizable to younger children. Further, only studies written in English were included which may exclude studies published in other languages, particularly from families of different ethnic backgrounds. The majority of studies also had small sample sizes (n < 100), and a lack of standardized assessment tools for outcomes measured (e.g., psychological flexibility). One of the most central changes that ACT targets is an increase in psychological flexibility, and other changes such as decreased depressive symptoms are often considered secondary outcomes [29], but only three of the ten reviewed studies used the AAQ-II to measure changes in psychological flexibility, so we could not compare this outcome across all the studies.

Specific limitations of the current meta-analysis include the small number of studies that were included in the analysis. This was largely due to the limited number of studies that met inclusion criteria and the variety of the outcomes reported by each study. One of the studies included in the meta-analyses had a high risk of bias; however, it was still included because of the small number of studies that met the inclusion criteria. Finally, there was considerable heterogeneity in the meta-analyses assessing stress, avoidance and fusion in children with SHCN and anxiety in parents of children with SHCN. 

## 5. Conclusions

Our findings highlight that ACT may help improve depressive symptoms in children with SHCN and psychological inflexibility in their parents. Emerging research on ACT for children with SHCN and their parents is encouraging. Future research on ACT in children with SHCN and their parents should include robust study designs a variety of conditions, larger sample sizes, and longer follow-up periods to determine the effectiveness of ACT. Improved treatment for conditions such as depression and anxiety in children with SHCN and their parents is an important priority for future research in order to understand ideal delivery models and opportunities for preventative and family-centered care.

## Figures and Tables

**Table 1:** Study Characteristics for ACT in Children with SHCN and Parents of Children with SHCN (n = 10).

Author, Year and Country/Region	Study Design	N	Age Range in years	Mean Age, years ± SD	% Female	Diagnosis	ACT Frequency and Delivery	Core ACT Processes	Length of Follow-Up	Instrument(s) Assessing Mental Health and Behavioural Outcome(s)	Instrument(s) Assessing ACT Related Outcome(s)/Process Variable(s)
**Children**											
Hancock 2018 [51] Australia	RCT	157	7–17	11 ± 2.76	58	Anxiety Disorders	10 weekly sessions, each session lasted 90 min with a psychologist	Acceptance, cognitive defusion, mindfulness, values, committed action and self-as-context	3 months post-ACT	-Anxiety Disorders Interview Schedule -Multidimensional Anxiety Scale for Children	Avoidance and Fusion Questionnaire for Youth
Swain 2015 [37] Australia	RCT	49	7–17	13.8 ± 1.4	63.3	Anxiety Disorders	10 weekly sessions, each session lasted 90 min with a psychologist	Acceptance, cognitive defusion, mindfulness, values, committed action and self-as-context	3 months post-ACT	-Child Behaviour Checklist -Children’s Depression Inventory	Not assessed
Ataie-Moghanloo 2015 [57] Iran	Pre-post with control group and random assignment	34	7–15	ACT: 10.35 ± 2.91 Control: 10.59 ± 3.16	50	Type I and II Diabetes	10 weekly sessions, each session lasted 90 min with a psychologist	Creative hopelessness, values, committed action, acceptance and control, cognitive defusion, self-as-context	None	-Reynolds’ Child Depression Scale	Not assessed
Moazzezi 2015 [58] Iran	Pre-post with control group and random assignment	36	7–15	ACT:11.44 ± 2.59 Control:9.72 ± 2.37	30.56	Type I and II Diabetes	10 weekly sessions, each session lasted 90 min with a psychologist	Creative hopelessness, values, committed action, acceptance and control, cognitive defusion, self-as-context	None	-Total Perceived Stress Scale	Not assessed
Azadeh 2015 [54] Iran	Quasi-experimental pre-post with random assignment	30	15–16	15.43 ± 0.78	100	Social Anxiety Disorder	10 weekly sessions, each session lasted 90 min with a psychologist	Not reported	None	-Interpersonal problems (assertiveness, sociability, submissiveness, intimacy, taking responsibility, and controlling)	-Acceptance and Action Questionnaire-II (measures psychological flexibility)
Ghomian and Shairi 2014 [55] Iran	Quasi-experimental pre-post with random assignment	20	7–12	ACT: 10.60 ± 1.7 Control:10.20 ± 1.81	ACT: 40 Control: 50	Chronic Pain	8 sessions with a psychologist	Creative hopelessness, values, cognitive defusion, acceptance and control, committed action	1.5 and 5 months post-ACT	-Child Behaviour Checklist	Not assessed
Livheim 2014 [52] Australia	RCT (only girls were randomized due to limited number of boys)	51	12.5–17.75	14.6 ± 1.03	63	Depressive Symptoms	8-week group program with a psychologist	Acceptance, cognitive defusion, mindfulness, values, committed action and self-as-context	None	-Depression, Anxiety and Stress Scale-21 -Perceived Stress Scale	-Avoidance and Fusion Questionnaire for Youth
Livheim 2014 [52] Sweden	RCT	32	14–15	Not Reported	71.8	Psychological Problems (Scoring > 80th percentile on Strengths and Difficulties Questionnaire)	8 group sessions were adapted to fit within a period of 6 weeks (90 min each) with psychologists	Acceptance, cognitive defusion, mindfulness, values, committed action and self-as-context	None	-Reynolds Adolescent Depression Scale	-Avoidance and Fusion Questionnaire for Youth
**Parents**											
Chong 2019 [53] Hong Kong	RCT	168	18–65 (parents) 3–12 (children)	38.40 (±5.90)	88	Asthma (children)	4 weekly sessions (90 min) delivered by nurse (first author) trained in ACT	Acceptance, cognitive defusion, mindfulness, values, committed action and self-as-context	6 months post-ACT	-Depression Anxiety Stress Scale-21	-Acceptance and Action Questionnaire-II
Hahs 2019 USA	Pre-post with control group and random assignment	18	34–57 (parents) 5–13 (children)	Parents: 45.5 (±6.14) Children: 8.44 (±2.52)	72.2	Autism spectrum disorders (children)	Two 2 h training sessions one week apart, delivered by the first author	Acceptance, cognitive defusion, mindfulness, values, committed action and self-as-context	One week post-session one	Not assessed	-Acceptance and Action Questionnaire-II (measures psychological flexibility)
Gharashi 2019 Iran	Quasi-experimental pre-post with random assignment	32	22–37 (parents)2–6 (children)	Intervention: 29.31 (±4.47) Control: 30 (±3.01)	100	Hearing impairment or deafness (children)	Eight 90-min sessions over 4 weeks, delivered by a therapist	Acceptance, cognitive defusion, mindfulness, values, committed action and self-as-context	Four weeks post-session one	-Depression Anxiety Stress Scale-21	Not assessed

RCT: Randomized Controlled Trial.

## Abbreviations

AAQ-II	Acceptance and Action Questionnaire-II
ACT	Acceptance and Commitment Therapy
CBCL	Child Behavior Checklist
CBT	Cognitive Behavioural Therapy
CI	Confidence Intervals
DASS-21	Depression, Anxiety and Stress Scale-21
SD	Standard Deviation
SMD	Standardized Mean Difference
